# Port Site Consequences After Laparoscopic Cholecystectomy Using an Open Versus Closed Approach of Pneumoperitoneum

**DOI:** 10.7759/cureus.26499

**Published:** 2022-07-01

**Authors:** Awni Ismail Sultan, Sami Hassoon Ali, Ozdan Akram Ghareeb

**Affiliations:** 1 Department of Surgery, College of Medicine, Tikrit University, Kirkuk, IRQ; 2 Department of Surgery, Jalawla General Hospital, Kirkuk, IRQ; 3 Department of Community Health, Northern Technical University, Kirkuk, IRQ

**Keywords:** hematoma, bleeding, cholecystectomy, pneumoperitoneum, laparoscopy

## Abstract

Introduction: Laparoscopic surgery is the standard method for cholecystectomy, and pneumoperitoneum is performed either in a closed or open technique. However, exposure to the consequences of the port site may increase the patient's morbidity. Therefore, this study was conducted to compare both approaches in terms of complications at the port site of each procedure and potential risk factors.

Methods: A prospective study was conducted in the department of surgery, in hospitals affiliated with Kirkuk and Diyala governorates in Iraq, from January 2019 to March 2022. The participating patients (200) were electively divided into two groups, each group comprising 100 patients. The pneumoperitoneum was established in the first group by an open technique (Hasson) while in the second group it was by using a closed technique (Veress needle). A comparison was made between the two techniques for intraoperative and postoperative complications that may have occurred due to port insertion up to 18 weeks.

Results: According to the results, the highest percentage was for the following: females (84.0%), ages between 50 and 59 years (43.5%), and body mass index (BMI) range 25-30 kg/m^2 ^(49.0%). No significant difference was observed between those variables for the two surgical techniques (p-value > 0.05). No death was recorded in the study. Consequences at the port site were observed in 10.5% of patients, the majority reported in the open approach (8.5%) as follows: bleeding (3.0%), hematoma (2.0%), wound infection (1.5%), hernia (1.5%), and vascular injury (0.5%).

Conclusions: Thus, we concluded that port site complications are lowest in closed laparoscopic surgery which was not shown to be statistically significant but values showed less complications. Furthermore, samples could be used to gain a good statistical significance.

## Introduction

Since the advent of laparoscopy in 1910 by Jacobus from Sweden, its utilization has made it superior to laparotomy in the management of many diseases [1,2]. Currently, this technology is widely used for diagnostic [3] and therapeutic purposes [4]. This technique allows the surgeon to enter the abdomen and pelvis by making a relatively small incision on the skin, known as keyhole surgery [5]. Its significant advantages include early recovery after surgery, less post-operative pain, and better cosmetic results [6]. However, the laparoscopic technique comes with risks [7]. So there may be consequences after laparoscopic surgery, and they usually occur while trying to access the peritoneal cavity [8]. It has been proven that more than half of the complications have happened at the time of insertion in most patients [9]. Consequences that may occur during the operation include visceral and vascular injuries and bleeding. As for the postoperative results, wound infection, hematoma, and hernia may occur at the port site during the follow-up period [10]. Today, laparoscopic cholecystectomy has become the most widely performed elective procedure and for its intelligible advantages, it has become the treatment of choice for symptomatic gallstones and cholecystitis, both acute and chronic [11-13]. Closed (Veress needle) and open (Hasson trocar) techniques are approaches for creating a pneumoperitoneum [14]. There are relatively few published studies to compare the advantages and disadvantages of each of them. No definitive superiority of any technique has been proven over the other [15], so choosing the most appropriate method depends on surgeons' cumulative experience [16]. The current study was done to assess the risk factors of these two techniques and the potential consequences for each surgical procedure.

## Materials and methods

Study design

This prospective, controlled study included 200 patients ( power of the study was calculated by the use of G power software to give the subject number based on the patient's availability and the number of cases in the region) with symptoms of cholecystitis who underwent elective laparoscopic cholecystectomy, which was performed in the general surgery departments in hospitals in Kirkuk and Diyala governorates (Iraq) by specialized laparoscopic surgeons during the period from January 2019 to March 2022. The study was completed after obtaining approval from the ethical committee of both Kirkuk and Diyala health directorates with a number (Ref 18/12/18). Cholelithiasis was diagnosed clinically as well as by abdominal ultrasonography.

Methodology

Every patient went through preoperative testing with specialists that specialize in that field. Anesthesiologists performed examinations on them before the operation. On the day of operation, the essential antibiotics were given intravenously, and this was immediately followed by a predetermined course of treatment with these antibiotics.The body mass index (BMI) was determined for every patient who took part in this study. All the patients who underwent laparoscopic procedures were divided into two groups A and B. In group A, pneumoperitoneum was created using the closed technique and in group B it was created using the open technique. Both the closed and open surgical techniques of laparoscopic cholecystectomy, which are the two primary techniques of intraperitoneal carbon dioxide (CO_2_) insufflation, are utilized in our laparoscopic centers. Parameters accessed as the demographic data of the patients were age, gender, and BMI of the patient at the baseline. After all of the patients had been given a general anesthetic, one of the two surgical procedures was performed according to the two groups. The early complications were also evaluated and were measured for a period of one month and then later on a single visit per month was kept for follow-up. The parameters at baseline were to categorize the subjects as they are not related to the surgery consequences. Various port site consequences were then recorded and analyzed. 

Inclusion and exclusion criteria

Patients, who underwent laparoscopic cholecystectomy, whether using an open or closed approach from pneumoperitoneum, were included of both sexes, and their ages ranged between 20 and 66 years. Patients who had skin disorders and co-morbid conditions such as diabetes, hepatitis, and others and previous abdominal diseases that prevented them from laparoscopic surgery were excluded from the current study.

Surgical procedure

The closed approach is via a special (Veress) needle. At the same time, the open method is performed by creating a small entrance directly into the peritoneal cavity under direct vision and is less used. The first (closed) procedure is more commonly performed because it is easier, takes less time, and the muscle sheath does not require closure at the end of the surgery. While the latter open approach is mainly suitable for any case of suspected intra-abdominal adhesions. Patients were discharged from hospitals within the first week after surgery and checked for immediate complications if were there and treated for the same. To assess port site consequences incidence, patients were followed up postoperatively for any undesirable effects for a period of one month. A monthly outpatient clinic check lasted around 18 weeks, with one examination per week in the first postoperative month and one visit per month for the remaining three months.

Statistical analysis

The data in this study were analyzed descriptively using the Statistical Package for the Social Sciences (SPSS) program (version 26, IBM Corp., Armonk, NY), the results were represented on numerical frequencies and percentages when tabulating the data, and appropriate non-parametric tests were applied to detect the significance of the study variables between study groups, with the assessment of the level of the value set at p < 0.05.

## Results

When the data of (200) patients who underwent laparoscopic cholecystectomy were analyzed, we found that (16.0%) of them were male and (84.0%) were female. About (9.0%) of the males and (41.0%) of the females underwent the open technique compared to (7.0%) and (43.0%), respectively, for the closed technique, as no clear difference was observed between the surgical techniques (p-value = 0.440). As for the age of patients, it was noted that the maximum age group was between 50 and 59 years old (43.5%), where it was 21.5% of them within the open group versus 22.0% for the closed group, while the minimum age group was between 60 and 69 years old (7.5%), as it has been proven that 4.0% of them are in the open group and 3.5% in the closed group without any clear statistical difference between surgical approaches (p-value = 0.992). The highest percentage of BMI was recorded (49.0%) between 25 and 30 kg/m^2^, 24.0% for the open group versus 25% for the closed one, while the lowest percentage (8.5%) was within <18.5 kg/m^2^ recorded. 5.0% are within the open group versus 3.5% for the closed group. Also, no considerable difference was observed between surgical approaches for this variable (p-value = 0.743) (Table 1).

**Table 1:** Characteristics of patients who underwent laparoscopic cholecystectomy. BMI: body mass index

Characteristics		Total N=200	Open approach N=100	Closed approach N=100	P-value
Gender	Male	32 (16.0%)	18 (9.0%)	14 (7.0%)	0.440
	Female	168 (84.0%)	82 (41.0%)	86 (43.0%)	
Age (years)	20-29	17 (8.5%)	9 (4.5%)	8 (4.0%)	0.992
	30-39	36 (18.0%)	17 (8.5%)	19 (9.5%)	
	40-49	45 (22.5%)	23 (11.5 %)	22 (11.0%)	
	50-59	87 (43.5%)	43 (21.5%)	44 (22.0%)	
	60-69	15 (7.5%)	8 (4.0 %)	7 (3.5 %)	
BMI (kg/m^2^)	<18.5	17 (8.5%)	10 (5.0% )	7 (3.5%)	0.743
	18.5-24.9	32 (16.0%)	14 (7.0%)	18 (9.0%)	
	25-30	98 (49.0%)	48 (24.0%)	50 (25 %)	
	>30	53 (26.5%)	28 (14.0%)	25 (12.5%)	

Among the 200 patients, port site consequences were observed in 21 (10.5%) patients, 17 (8.5%) of them in the open approach group and four (2.0%) in the closed approach. Bleeding was the most frequent occurrence as nearly eight (4.0%) of the total patients developed bleeding; six (3.0%) of them were in the open approach group, while two (1.0%) of them were in the closed approach group (p-value = 0.149). Followed by the wound infection at the port site, as five (2.5%) cases were recorded, three (1.5%) in the open group versus two (1.0 %) in the closed one (p-value = 0.651). Whereas four (2.0%) developed a hematoma at the port site, and all of them had the open technique with a significant difference (p-value = 0.043) from the closed technique group (0.0 %). As for the port site hernia, three (1.5 %) cases were recorded in the open group (p-value = 0.081). Also, only one case (0.5%) for vascular injury was recorded in the closed group (p-value = 0.316). No visceral injury occurred in both approaches (Table 2).

**Table 2:** Comparison of port site consequences in both surgical approaches.

Consequences	Total N=200	Open approach N=100	Closed approach N=100	P-value
Vascular injury	1 (0.5 %)	1 (0.5 %)	0 (0.0 %)	0.316
Visceral injury	0 (0.0 %)	0 (0.0 %)	0 (0.0 %)	/
Bleeding	8 (4.0 %)	6 (3.0 %)	2 (1.0 %)	0.149
Port site wound infection	5 (2.5 %)	3 (1.5 %)	2 (1.0 %)	0.651
Port site hematoma	4 (2.0 %)	4 (2.0 %)	0 (0.0 %)	0.043
Port site hernia	3 (1.5 %)	3 (1.5 %)	0 (0.0 %)	0.081
Total	21 (10.5 %)	17 (8.5 %)	4 (2.0 %)	/

## Discussion

Laparoscopic surgery has several advantages over laparotomy due to the small incision, faster recovery, and enabling the surgeon to better view and magnify the anatomy of the abdominal cavity [17]. One of the main steps in laparoscopic cholecystectomy is the induction of pneumoperitoneum, usually accompanied by hemodynamic and respiratory adverse effects. However, these negative changes can be managed and controlled [18,19]. Complications that the patient may encounter during or after laparoscopic surgery are still a problem faced by the surgeons [20]. In an attempt to avoid port site consequences, other techniques have been developed in practice, including the open technique devised by Harrith M. Hasson (1974), who described the open method of pneumoperitoneum to eliminate complications associated with the port entry. Still, this approach has not gained much acceptance because it has been reported to be time-consuming and associated with a large gas leaks [20-22]. In this study, females had more patients than males, which aligned with most of the previous literature [23,24]. It is worth noting that previous studies demonstrated that being overweight was associated with increased port site-related morbidity due to various factors such as the requirement for longer trocars, the thickness of the abdominal wall, and the necessity for a larger skin incision, as increased subcutaneous tissue causes a limitation of instrument movement [25]. The current study showed that laparoscopic cholecystectomy was associated with port site complications with a rate of up to (10.5%), with the highest rate of bleeding cases (4.0%), followed by wound infections (2.5%), and then hematoma (2.0%). Our results are comparable with Radunovic and colleagues (2016) study when they analyzed the medical records of 740 patients who underwent laparoscopic cholecystectomy retrospectively, of whom (13.1%) had experienced complications during the procedure. Bleeding (3.64%) was the most common, and (0.94%) surgical wound infection [26]. At the same time, a recent study conducted by Alam and colleagues (2021) found that among 108 patients who underwent laparoscopic cholecystectomy, 12 (11%) of them developed a port site infection [27]. When comparing those complications between both surgical techniques, we noticed that (8.5%) were in the open group versus (2.0%) in the closed group. This was in agreement with Nawaz and his colleagues (2016) results when they compared the closed and open laparoscopic cholecystectomy techniques in terms of complications for 140 patients. They noticed that (1.3%) of the patients suffered from postoperative hematoma and (2.6%) had an infection at the surgical site, and they were from the open group only. No complications were observed in the closed technique [28]. Our results were also supported by a study conducted by Manjunath and Jayaprakas on 90 patients who underwent laparoscopic surgery, who noted that fewer complications characterized the Veress needle method than the open technique [29].

The study's limitations can be in the form of limited samples being studied, and so also, there have been just two approaches being studied. Various other methods could have also been studied. Various complications also could be incorporated into the study parameters. So also, due to ease of surgery surgeons likely may have chosen an open approach because of the previous history of abdominal surgery which could be a bias..

## Conclusions

According to the findings of the current study, both open and closed approaches to accessing the peritoneal cavity can be considered safe. The values are not statistically significant in both approaches. A closed and open approach could be used simultaneously according to the surgeon's preference. Hence the study has added to the literature on the safety of the use of both approaches for accessing the peritoneal cavity.
